# Knowledge and Use of Artificial Intelligence Among Oncology Faculty and Trainees at a Comprehensive Cancer Center in 2025

**DOI:** 10.1200/CCI-25-00300

**Published:** 2026-05-27

**Authors:** Keri Schadler, Ann Klopp, Ramez Kouzy, Pavitra P. Krishnamani, Rebekah Filson, Aiden Moody, Elizabeth Grubbs, Nicole Munoz, Lauren Payne, Shawn Stapleton, Caroline Chung

**Affiliations:** ^1^ Department of Pediatrics Research, The University of Texas MD Anderson Cancer Center, Houston, TX; ^2^ Department of Radiation Oncology, The University of Texas MD Anderson Cancer Center, Houston, TX; ^3^ Department of Emergency Medicine, The University of Texas MD Anderson Cancer Center, Houston, TX; ^4^ Department of Academic Affairs, The University of Texas MD Anderson Cancer Center, Houston, TX; ^5^ New York University, New York, NY; ^6^ Institute for Data Science in Oncology, The University of Texas MD Anderson Cancer Center, Houston, TX

## Abstract

**PURPOSE:**

Artificial intelligence (AI) has been used in medicine for decades, but recent advances in machine learning and large language models have rapidly expanded its accessibility and applications in oncology. Although AI offers efficiency in data analysis, synthesis, and communication, important questions remain regarding output reliability, research rigor, data security, and appropriate reliance on automated tools, underscoring the need for thoughtful implementation and training.

**METHODS:**

To better understand current AI use in academic oncology and perceptions about utility, we conducted a survey of faculty and trainees at a comprehensive cancer center.

**RESULTS:**

Among 227 respondents (55% women; 64% clinical faculty), 58% reported using AI several times per month or more, whereas 15% had never used it. The most common applications were summarizing academic or research information and generating data visualizations. Attitudes toward AI were generally positive: 74% agreed that AI will improve cancer diagnosis within the next decade. In contrast, views were more cautious regarding end-of-life decision making, with 35% disagreeing that AI would be beneficial in that context. Despite broad interest, a substantial training gap emerged. Nearly 93% of respondents endorsed the need for dedicated AI training, and approximately half reported not knowing where to find reliable learning resources. Lower AI use was associated with female gender and age over 60 years.

**CONCLUSION:**

AI use among oncology faculty and trainees is common but variable, with differences across demographic and professional groups. These findings highlight the need for structured, accessible training and institutional guidance to promote appropriate, equitable, and high-quality integration of AI into oncology research and clinical care.

## Graphical abstract

**Figure:**
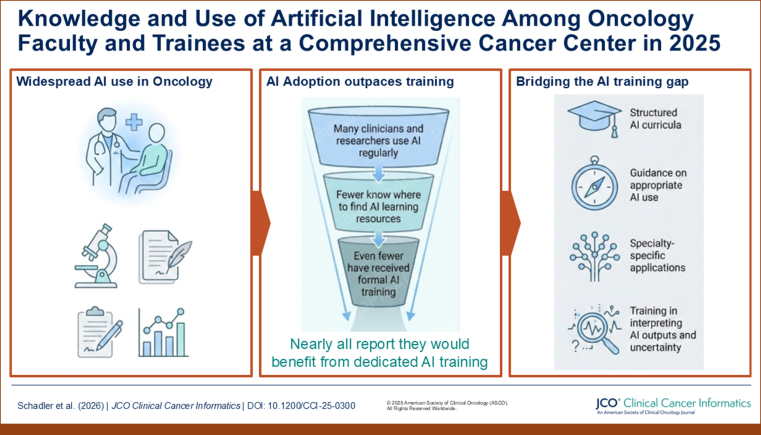

## INTRODUCTION

The integration of artificial intelligence (AI) into oncology practice has accelerated dramatically in recent years. Although AI-based tools have long supported diagnostic imaging and radiation oncology workflows,^1^ the current wave of innovation has expanded into ambient clinical documentation, code generation for data analysis, and generative writing for a broad range of academic applications.^2^ Academic institutions face unique challenges in this evolving landscape. The rapid pace of AI advancement, coupled with unclear or shifting policies, creates uncertainties around best practices for education, oversight, and deployment. This makes it exceedingly difficult to provide guidance and education to optimize AI use by maximizing organizational AI capabilities while mitigating risks. To enable both education and usage policy development for our institution, we sought to understand the current patterns of use and attitudes toward AI by clinical and research faculty and trainees at our institution through a brief online survey. Our goal was to characterize patterns of AI usage, assess perceptions of utility and risk, and identify gaps and interests in training modalities.

**CONTEXT**

**Key Objective**
What are the patterns of artificial intelligence (AI) use, knowledge, and training needs among faculty and trainees at a comprehensive cancer center, and how can these insights guide responsible academic integration of AI?
**Knowledge Generated**
We found widespread but uneven AI adoption across clinical and research roles, with use spanning writing, summarization, coding, and data analysis and with many respondents reporting unmet needs for structured education and institutional guidance. Despite these gaps, participants expressed strong optimism that AI will improve cancer diagnosis, cancer treatment, management of treatment side effects, clinical and preclinical research efficiency, and clinical faculty's skills and reduce disparities in cancer care.
**Relevance *(D. S. Bitterman)***
Oncology faculty and trainees see clear opportunity to enhance cancer care and research with AI. Efforts to improve AI literacy and appropriate use will be needed for these opportunities to come to fruition.*
*Relevance statement written by *JCO CCI* Associate Editor Danielle S. Bitterman, MD.

## METHODS

A cross-sectional, online survey consisting of 16 questions was used (Data Supplement, Fig S1). Participant responses were gathered through convenience sampling by sharing the invitation to take the survey through mass e-mails, by request during online or in-person meetings, and through personal communications at MD Anderson Cancer Center between April 24, 2025, and June 19, 2025. The survey was completed by 227 respondents who were either faculty or trainees engaged in oncology-related patient care, clinical research, and/or preclinical research. Administrative and nonfaculty staff were not included in the survey requests. The Clinical Institutional Review Board (IRB1) judged the project to be exempt as quality improvement/educational research.

The survey was modified based on a previously published survey^3^ related to ethical implications of AI use in cancer care, with a primary goal to identify the current usage patterns of AI and opportunities for education related to AI among faculty and trainees at MD Anderson Cancer Center. The survey included domains of demographics, AI-usage frequency, AI-task prevalence, attitudes toward AI, and AI-specific training (Data Supplement, Fig S1). Responses were grouped for analysis as follows: Age groups were collapsed into <40, 40-59, and ≥60 years. Role responses were recoded into two orthogonal variables, clinical versus research and faculty versus trainee. For AI-task prevalence, we retained every checklist option mentioned by ≥20 respondents (six of six task options met this threshold).

Survey results were reported with descriptive statistics. ChatGPT (OpenAI) was used as an interactive analytic aid during the exploratory phase to help surface potential patterns and guide analytic planning. All quantitative analyses were then executed in Python 3.14.2 using standard scientific computing libraries. Descriptive statistics included counts and column percentages for demographics, AI-usage frequency, and each AI task. Comparative analyses were performed using Pearson χ^2^ tests to examine associations between each demographic (Gender, Age group, Clinical *v* Research, Faculty *v* Trainee) and overall AI-usage frequency, binary indicators for each AI task, and binary agree indicators for the six optimism/trust statements and three training/resource items.

## RESULTS

The 227 respondents included 126 women (55%), 100 men (43%), and one person (2%) who did not identify as male or female. Of these, 146 (64.3%) were clinical faculty, 55 (24.2%) research faculty, 12 (5.3%) clinical trainees, and 14 (6.2%) research trainees. Trainees included residents, clinical fellows, graduate students, and postdoctoral fellows. The majority of respondents were between age 40 and 59 years (n = 117; 51.5%), with 39.2% <40 (n = 89) and 9.2% (n = 21) ≥60 years (Table 1).

**TABLE 1.** Survey Participant Characteristics (N = 227)

Survey Participants	No.	%
Age		
Less than 40 years	89	39.2
40-59 years	117	51.5
60-80 years	20	8.8
≥81 years	1	0.4
Gender		
Female	126	55.5
I do not identify as male or female	1	0.4
Male	100	44.1
Role		
Clinical	146	64.3
Research	55	24.2
Trainee	26	11.5

**FIG 1.**
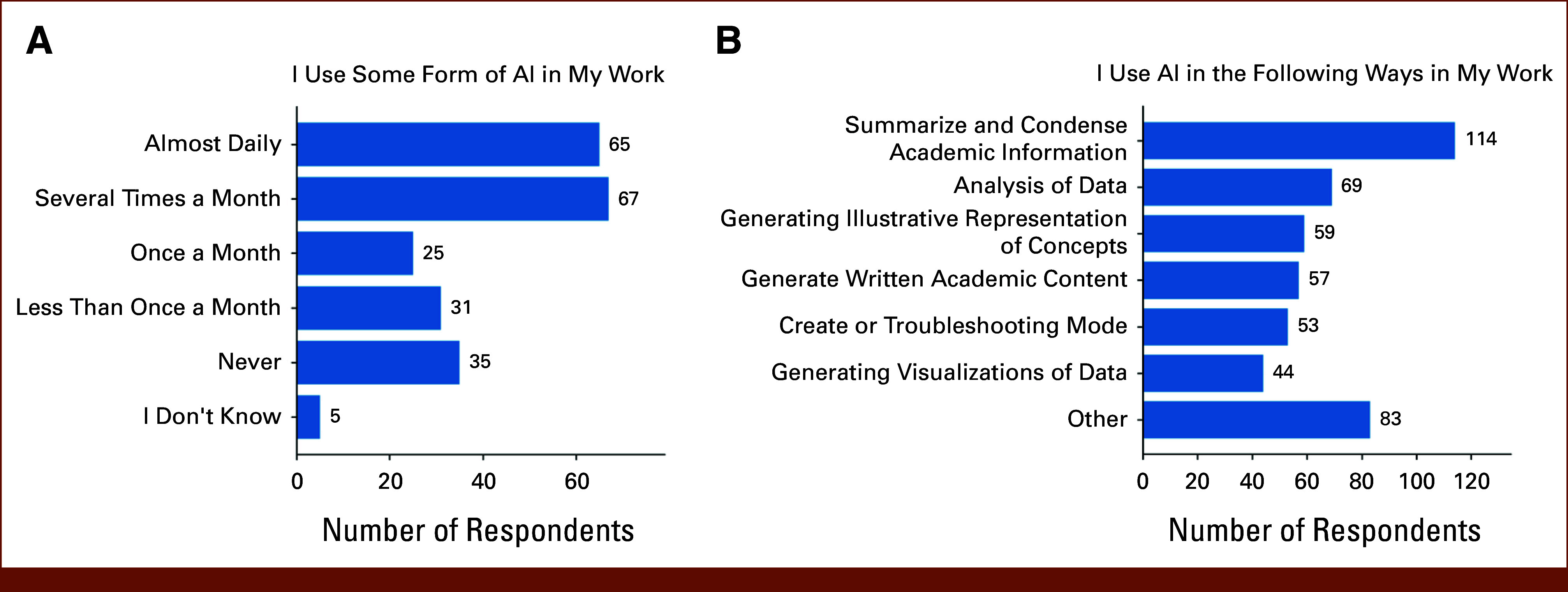
Frequency of AI use in clinical and research work. Distribution of self-reported AI use frequency among oncology faculty and trainees (n = 227) (A). Bars represent the number of respondents selecting each category. Most respondents reported using AI several times a month or almost daily, whereas a smaller proportion reported rare or no use. Distribution of use cases for AI in work (B). The most common applications included summarizing academic information, generating written academic content, data analysis, and code troubleshooting. Fewer respondents reported using AI for generating visualizations or illustrative concepts. Respondents could select more than one category. AI, artificial intelligence.

Most respondents (58%) used AI several times per month or almost daily, although 15% of respondents reported never using AI (Fig 1A). The most common tasks that AI is used for by respondents were to summarize academic or research information and to analyze data (Fig 1B). Among respondents selecting Other for AI use (54/228, 23.7%), free-text responses commonly described administrative and professional communication tasks (e-mails, letters of recommendation/reference, insurance/peer-to-peer letters, and editing/summarizing; 22/228, 9.6%), educational support such as study outlines and flashcards (10/228, 4.4%), and AI development or research workflows (9/228, 3.9%). Additional uses included imaging/vision AI embedded in clinical tools (eg, ultrasound enhancement and digital image analysis 8/228, 3.5%), clinical documentation support (4/228, 1.8%), radiation oncology planning/contouring (4/228, 1.8%), and clinical decision support/trial eligibility (4/228, 1.8%). Themes were derived from qualitative review of free-text responses and were not mutually exclusive

**FIG 2.**
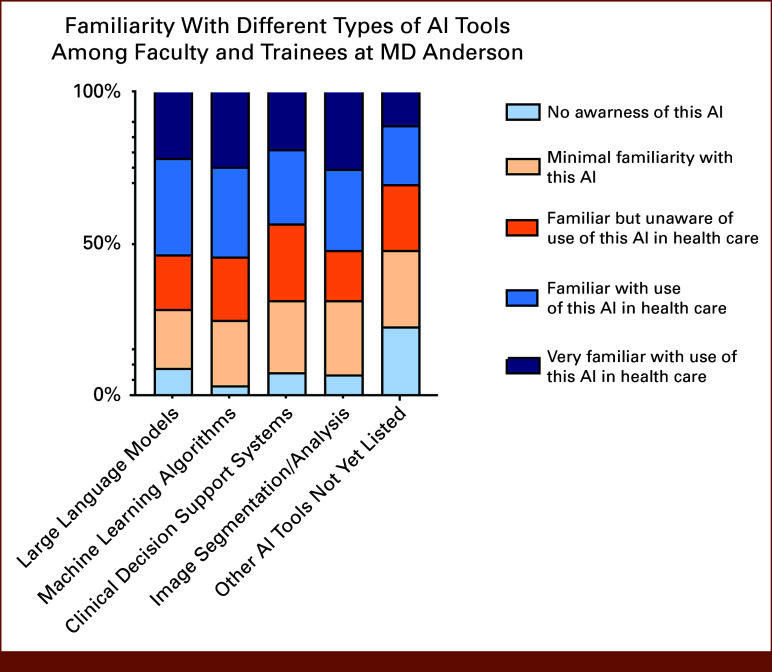
Familiarity with different types of AI tools among faculty and trainees. Stacked bar charts show the distribution of self-reported familiarity with several categories of AI tools, including large language models, machine learning algorithms, clinical decision support systems, image segmentation/analysis tools, and other AI tools. For each tool category, respondents selected one of five familiarity levels: no awareness of this AI, minimal familiarity, familiar but unaware of use in health care, familiar with use in health care, or very familiar with use in health care. Bars are displayed as percentages within each AI tool category and sum to 100%. AI, artificial intelligence.

**FIG 3.**
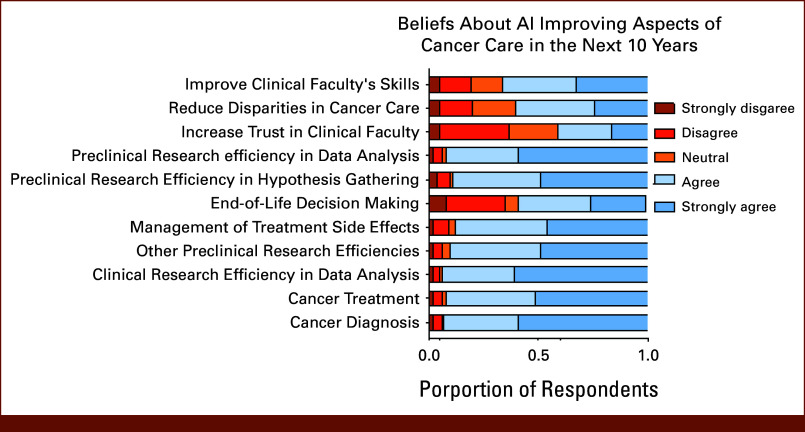
Beliefs about the potential impact of AI on aspects of cancer care over the next 10 years. Stacked bar charts show the distribution of responses regarding whether AI is expected to improve specific domains of cancer care and research. Response options ranged from strongly disagree to strongly agree. Respondents were most optimistic about AI improving efficiencies in preclinical and clinical research, data analysis, cancer diagnosis, and treatment. In contrast, expectations were more mixed for AI's role in increasing patient trust, reducing disparities in cancer care, improving clinical faculty skills, and supporting end-of-life decision making. AI, artificial intelligence.

The majority of respondents could think of an example of or were familiar with the use of large language models, machine learning algorithms, clinical decision support systems, and image segmentation/image analysis tools (Fig 2). Attitudes toward AI were largely positive, with the majority of respondents agreeing or strongly agreeing that in the next 10 years, AI will improve all categories listed in the survey. For example, 74% of respondents agreed/strongly agreed that AI will improve cancer diagnosis in the next 10 years. Less optimism was reported for end-of-life decision making, where 35% of survey respondents disagreed or strongly disagreed with the statement that AI will improve end-of-life decision making (Fig 3).

**FIG 4.**
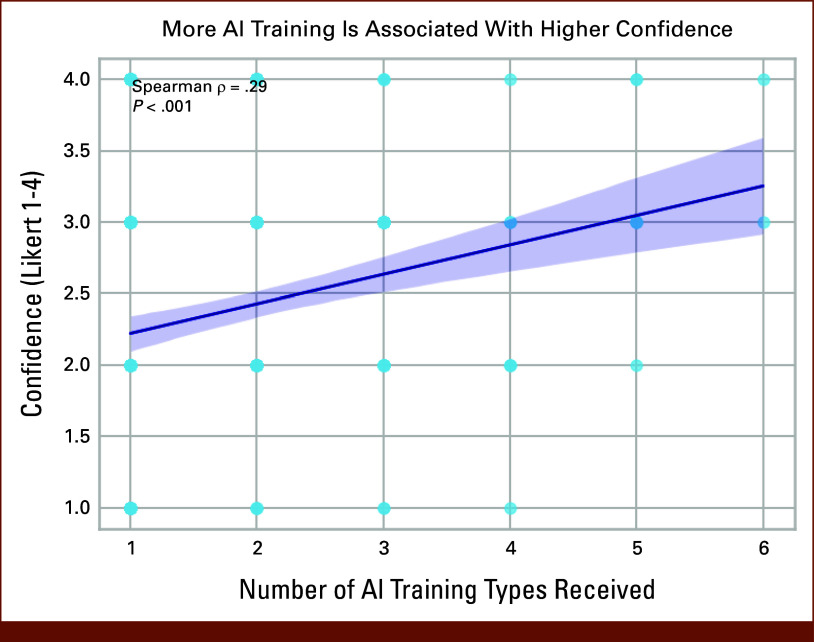
Association between AI training and confidence in using AI relationship between the number of different types of AI training received and self-reported confidence in using AI tools among survey respondents. Each point represents an individual respondent's confidence rating on a four-point Likert scale (1 = not confident, 4 = very confident). The solid line represents the fitted linear regression, and the shaded area indicates the 95% confidence interval. Greater exposure to AI training types was associated with higher confidence in using AI (Spearman ρ = 0.29, *P* < .001). AI, artificial intelligence.

The survey identified a need for training, with 93% of respondents affirming that they would benefit from dedicated training on AI for research or clinical care. The majority of respondents reported self-learning as their primary mode of training on AI, although half reported a lack of knowledge of where to find resources to teach themselves about AI. A clear gradient was observed between training intensity and confidence: respondents with self-reported advanced degrees (masters or doctorate) in AI reported the highest confidence in evaluating data set representativeness (92%), compared with 71%-70% among those with formal courses or certificate programs and just 27% among those without AI training. The number of types of training regarding AI usage that participants have received was positively associated with the confidence using AI (Spearman ρ = 0.29, *P* < .0001, Fig 4).

**FIG 5.**
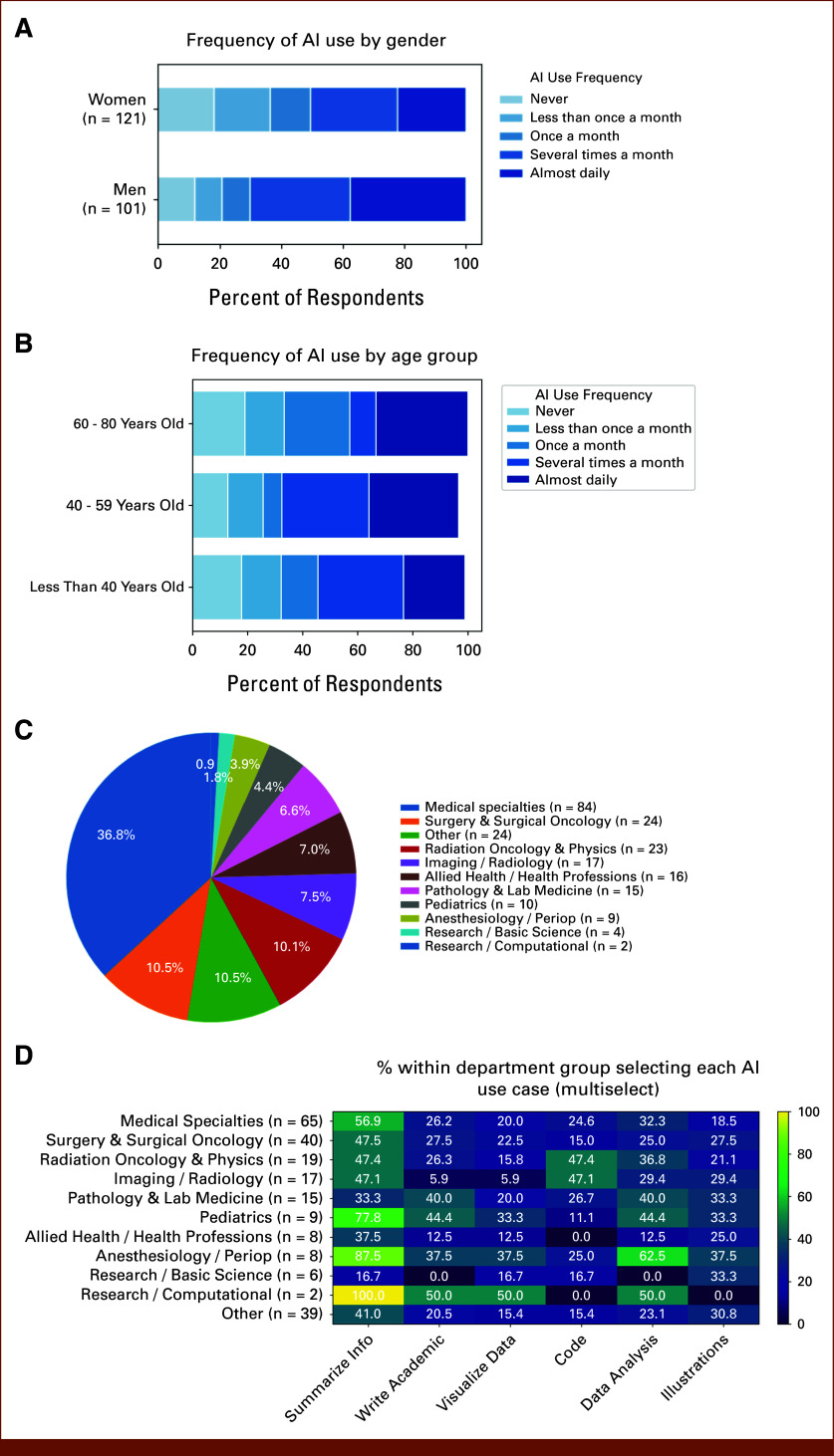
Frequency of AI use by gender, age and role. Stacked horizontal bar chart showing the proportion of respondents within each gender category reporting different frequencies of AI use (A). Men reported slightly higher proportions of almost daily AI use, whereas women showed a broader distribution across moderate-frequency categories. Sample sizes: men (n = 101), women (n = 121). Percentages may not total 100 due to rounding. Stacked horizontal bar chart illustrating AI use frequency stratified by age category (B). Younger respondents (<40 years) reported higher proportions of frequent AI use compared with older age groups, although substantial AI adoption was observed across all ages. Age categories were consolidated as <40 years (n = 90), 40-59 years (n = 117), and 60-80 years (n = 21). Percentages are calculated within age group. The distribution of self-reported job roles are shown in Figure 4C. Heat map showing the percentage of respondents within each department group selecting specific AI use cases (multiselect) (D). Warmer colors indicate higher within-group adoption of a given use case. Research-oriented and computational groups reported the highest use of coding and data analysis applications, whereas clinical specialties more commonly reported summarization and written content generation. Percentages are calculated within department group.

Respondents differed significantly in reported AI use by gender (Fig 5A). Among women, 61 (48%) were frequent users of AI (used it several times a month or almost daily), whereas among men, 70 (70%) were frequent users (χ^2^ = 9.8, *P* = .0017; Fig 5A). Females were more likely to use AI to summarize academic information (χ^2^ = 8.2, *P* = .017, V = 0.19). Males were significantly more likely than females to use AI for coding (χ^2^ = 12.6, *P* = .002, V = 0.24) and data analysis (χ^2^ = 8.8, *P* = .012, V = 0.20). Similarly, trainees were more likely than faculty, and researchers more likely than clinicians, to use AI for coding.

The frequency of AI use varied across age groups (Fig 5B). Participants younger than 40 years reported the highest levels of regular AI use, with a substantial proportion using AI several times per month or almost daily. Respondents age 40-59 years demonstrated a similar pattern, although with a slightly greater proportion reporting less frequent use. In contrast, participants age 60-80 years were more likely to report infrequent use or no AI use, and a smaller proportion reported near-daily use compared with younger groups. Across all age categories, however, a majority of respondents reported using AI at least several times per month. Younger people were more likely to use AI for data analysis (<40 *v* ≥ 40 years; χ^2^(4) = 11.6, V = 0.23).

In addition to a relationship between training type and AI usage, we observed differences in how AI is used based on specialty and job role. Participants represented several different academic affiliations (Fig 5C). Patterns of AI use varied across departments (Fig 5D). Academic writing and information summarization were common across many clinical specialties, whereas coding-related uses were more frequently reported in technically oriented fields such as Radiation Oncology & Physics and Imaging/Radiology (Fig 5D). Data analysis applications were also more prevalent in Anesthesiology/Perioperative Medicine, Pediatrics, Pathology & Laboratory Medicine, and Radiation Oncology & Physics.

In multivariable logistic regression adjusting for age, gender, and clinical versus research role, female gender was associated with lower odds of frequent AI use (aOR, 0.39, 95% CI, 0.22 to 0.69), and age ≥60 years was associated with lower odds compared with ages 40-59 (aOR, 0.29, 95% CI, 0.11 to 0.78). Clinical versus research role was not independently associated with frequent AI use (aOR, 1.22, 95% CI, 0.67 to 2.23)

## DISCUSSION

In this study, we found that the majority of faculty and trainee respondents of our survey use AI at least once per month, and many use it almost daily. Although there was wide variability in the tasks that AI is used for, the most common task that AI is used for is summarizing academic or research information. It is interesting that although attitudes toward AI were overall positive, there was still uncertainty about whether AI can improve end-of-life decision making or trust in physicians, indicating that physicians and researchers may be more instinctually hesitant to use AI in situations with subjective human emotion.

This finding, wherein participants viewed AI favorably in a data interpretation role but were wary of it replacing human empathy and expertise, is in line with the trend revealed in a 2021 review article examining multidisciplinary stakeholder attitudes toward AI in health care. Here too, stakeholder attitudes toward AI were generally found to be positive. However, AI that automated data interpretation and analysis was viewed in a more positive light by clinicians and consumers than AI that directly influenced clinical decision making or had the potential to affect the physician-patient relationship. Interestingly, this review also found that clinicians in pathology and radiology saw the greatest potential of the use of AI in their work compared with clinicians from nonimaging-based disciplines who viewed AI's use as more limited in their practice.^4^

The sentiment is shared globally, with the vast majority of surveyed general practitioners in the United Kingdom believing that human empathy and communication could not be replaced by AI in one study. It was also found that the majority of surveyed participants also believed that AI could not replace a clinician's role in decision making, particularly where value-based care was required.^5^ Another study, surveying psychiatrists across 22 countries, found that they felt AI was best suited to organize, update, and synthesize information when used in a health care setting.^6^ A study in Korea also found that physicians overwhelmingly considered AI to be useful in real-time clinical data analysis, but mirrored a similar sentiment with regard to medical decision making, with several expressing concerns that AI would not be effective in more obscure clinical scenarios given the lack of information available to train on in these situations.^7^

In surveying MD Anderson faculty, we identified both high enthusiasm for AI in biomedical research and health care and a clear need for better education to enable AI use. Virtually all respondents agreed that they would benefit from training on how to use AI, but half did not know how to find education or training on their own. This suggests that, in addition to advanced or task-specific training, many faculty and trainees would benefit from foundational, introductory education about AI.

In addition to widespread enthusiasm for training, the content and focus of such education are critical. A tiered curriculum could begin with foundational introductions to AI concepts, followed by discipline-specific applications such as research analysis, coding, and document summarization. Importantly, training should emphasize source verification and the recognition of limitations inherent to AI-developed models, particularly given the risks of inaccurate or hallucinated outputs from large language models. Developing practical data literacy skills, such as understanding appropriate use cases, recognizing uncertainty in outputs, and knowing when independent validation is needed, will help faculty and trainees critically evaluate AI-generated information. Embedding these competencies within faculty development and graduate medical education programs would not only accelerate responsible adoption of AI but also safeguard against misuse or overreliance on automated systems. Equally important, training must address the needs of the current workforce to ensure quality, safety, and regulatory compliance when using AI and data-driven tools. These efforts must be complemented by appropriate organizational infrastructure so that individuals can act on this training. At MD Anderson, we have built and continue to mature a data and AI governance framework that incorporates education and training as key risk management controls when deploying AI-based solutions. Beyond training, surveys such as ours can help institutions anticipate how individuals are exploring this technology and where risks may arise, which in turn help inform enterprise governance processes and safe adoption of AI tools.

This desire for more training also mirrors global sentiment around the use of AI in health care settings, with clinician liability being one of the top concerns expressed by stakeholders in this area. In the review article, training curricula, guidelines, and the development of working groups with AI expertise were desired to better prepare clinicians to introduce AI into their practice. This was true of those in independent practice as well as medical students and physicians in training, who desired more training in AI.^4^ The specific type of AI training was not addressed in the survey, especially regarding the use of clinical tools. Future analysis of the most effective training tools for diverse and evolving applications will be needed.

The limitations of this study include the convenience method of survey distribution. The total number of faculty and trainees who had access to the survey was approximately 2000 faculty and trainees, so approximately 10% of those eligible took the survey. Notably there were more faculty than trainees who completed the survey. These results may then reflect a bias in respondents with individuals with prior interest in or experience using AI may have been more likely to respond, although the responses demonstrate that many nonusers also completed the survey. Accordingly, these findings should be interpreted as descriptive of AI use among respondents rather than representative of all clinicians and researchers at our institution or elsewhere. Furthermore, AI use is rapidly expanding, and the results reported here are likely to underrepresent current usage. Additionally, there are not sufficient numbers to determine whether differences in attitudes toward AI by gender are truly gender differences or are driven by unbalanced numbers between specialties which include majority male or female faculty and also have high numbers of AI users (such as radiation oncology).

AI use among oncology faculty and trainees is common but variable, with differences across demographic and professional groups. Overall, our faculty and trainees reported a high level of optimism around the positive impact of AI tools in oncology but reported a strong need for more training. These findings highlight the need for structured, accessible training and institutional and societal guidance to promote appropriate, equitable, and high-quality integration of AI into oncology research and clinical care.
